# The N Terminus Specifies the Switch between Transport Modes of the Human Serotonin Transporter*

**DOI:** 10.1074/jbc.M116.771360

**Published:** 2017-01-19

**Authors:** Carina Kern, Fatma Asli Erdem, Ali El-Kasaby, Walter Sandtner, Michael Freissmuth, Sonja Sucic

**Affiliations:** From the Institute of Pharmacology, Center of Physiology and Pharmacology, Medical University of Vienna, A-1090 Vienna, Austria

**Keywords:** neurotransmitter release, neurotransmitter transport, patch clamp, serotonin, serotonin transporter

## Abstract

The serotonin transporter (SERT) and other monoamine transporters operate in either a forward transport mode where the transporter undergoes a full transport cycle or an exchange mode where the transporter seesaws through half-cycles. Amphetamines trigger the exchange mode, leading to substrate efflux. This efflux was proposed to rely on the N terminus, which was suggested to adopt different conformations in the inward facing, outward facing and amphetamine-bound states. This prediction was verified by tryptic digestion of SERT-expressing membranes: in the absence of Na^+^, the N terminus was rapidly digested. Amphetamine conferred protection against cleavage, suggesting a relay between the conformational states of the hydrophobic core and the N terminus. We searched for a candidate segment that supported the conformational switch by serial truncation removing 22 (ΔN22), 32 (ΔN32), or 42 (ΔN42) N-terminal residues. This did not affect surface expression, inhibitor binding, and substrate influx. However, amphetamine-induced efflux by SERT-ΔN32 or SERT-ΔN42 (but not by SERT-ΔN22) was markedly diminished. We examined the individual steps in the transport cycle by recording transporter-associated currents: the recovery rate of capacitive peak, but not of steady state, currents was significantly lower for SERT-ΔN32 than that of wild type SERT and SERT-ΔN22. Thus, the exchange mode of SERT-ΔN32 was selectively impaired. Our observations show that the N terminus affords the switch between transport modes. The findings are consistent with a model where the N terminus acts as a lever to support amphetamine-induced efflux by SERT.

## Introduction

Synaptic transmission is terminated by rapid clearance of the neurotransmitter from the synaptic cleft. Serotonin (5-HT)^3^ is retrieved by the plasmalemmal serotonin transporter (SERT; SLC6A4) into the presynaptic terminals and subsequently concentrated in synaptic vesicles by the vesicular transporters (vMAT1/2, SLC18A1, and SLC18A2) of serotonergic neurons (1). The closely related transporters for dopamine (DAT; SLC6A3) and norepinephrine (NET; SLC6A2) and SERT form the subfamily of monoamine transporters within the solute carrier 6 family of Na^+^/Cl^−^ symporters. Monoamine transporters can operate in two modes. (i) In the forward transport mode, the transporter completes a conformational cycle, which is initiated by binding of the substrate (*e.g.* 5-HT in the case of SERT) and co-substrate ions Na^+^ and Cl^−^ to the outward facing conformation. The subsequent closure of an outward gate shields substrate and co-substrate in the occluded state, and the opening of an inner gate creates an exit pathway for release of Na^+^ and substrate. Binding of K^+^ (or of H^+^) to the resulting inward facing conformation drives the return step to the outward facing conformation (2–4). The forward transport mode allows for substrate accumulation. (ii) Amphetamines drive the exchange mode where the transporter undergoes the same conformational transitions steps until it reaches the inward facing conformation, but it does not complete the cycle. Instead, after release of the amphetamine, the transporter binds the endogenous monoamine and Na^+^ in the cytosol and returns to the outward facing state in the substrate-bound state. In the exchange mode the transporter seesaws through half-cycles, resulting in monoamine efflux (5). Exchange diffusion was proposed some 40 years ago as the mechanism of action of amphetamines (6). Sustained activation of substrate efflux is contingent on the rapid back-diffusion of amphetamines through the membrane (7). Finally, in the presence of high concentrations of high internal concentrations of Na^+^ and substrate, monoamine transporters can also operate in a third mode to sustain net efflux; this presumably relies on the reversal of the complete transport cycle.

Amphetamine-induced monoamine efflux also depends on (i) additional signals, the most prominent of which is phosphorylation by calmodulin-dependent kinase and/or protein kinase C (8–15); (ii) the lipid composition of the membrane, in particular the presence of PIP_2_ (16–18); (iii) binding of syntaxin 1A to the transporter (19); and (iv) the oligomeric state of the transporter (10). The decision to switch from the forward transport mode and the exchange mode requires a communication between the hydrophobic core of the transporter where substrates are bound and the intracellular segments of the transporter, which receive the input by *e.g.* phosphorylation or binding of PIP_2_ or syntaxin 1A. Previous experiments show an important role of the N terminus in supporting amphetamine-induced monoamine efflux in SERT and DAT (20). The conjecture that the N terminus can act as lever is consistent with recent computational modeling (18). In the present study, we designed experiments to examine whether the conformation of the N terminus of SERT was modified by the occupancy of the binding site by amphetamines and, conversely, whether truncation of the N terminus affected the propensity of SERT to switch between the forward transport mode and the exchange mode. Our findings are consistent with the lever hypothesis and allow for a unified model where the role of the N terminus is to impose a switch in the kinetic cycle of SERT.

## Results

### 

#### 

##### Amphetamine-induced Conformational Change in the N Terminus of SERT Detected by Tryptic Digestion

**FIGURE 1.**
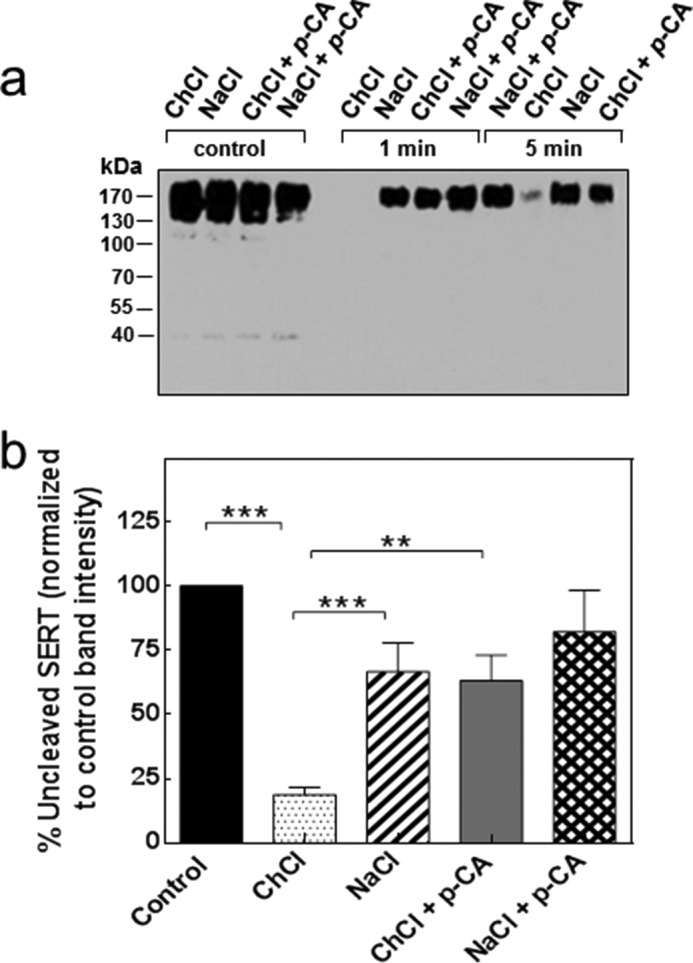
**Tryptic cleavage of the N terminus of SERT in the absence and presence of amphetamine.** Membranes (10 μg) of HEK293 cells stably expressing human YFP-SERT were incubated in buffer containing 150 mm choline chloride (*ChCl*) or 150 mm NaCl in the absence and presence of 10 μm *p*-CA at 22 °C. The reaction was started by the addition of 1.6 μg of trypsin and stopped by the addition of soy bean trypsin inhibitor (8 μg) after 1 or 5 min as indicated; the *first four left-hand lanes* (labeled *control*) represent the uncleaved starting material at *t* = 0. The cleavage pattern was analyzed by immunoblotting (*a*) as outlined under “Experimental Procedures” using an antibody recognizing the N-terminal YFP tag. The positions of molecular mass markers are indicated (in kDa). *b*, band intensities from 10 independent experiments (at *t* = 1 min) were quantified and expressed as a percentage of band density at *t* = 0. *Error bars* indicate S.E. The statistical comparison was done by analysis of variance followed by Tukey's multiple comparisons (***, *p* value <0.001; **, *p* value <0.01).

In the alternating access model, the transport cycle of SERT is linked to a conformational cycle where the hydrophobic core switches from an outward to an inward facing conformation. We surmised that this switch also affected the conformation of the extended N terminus. This conjecture was examined by subjecting SERT to limited proteolysis under different ionic conditions. Membranes were prepared from HEK293 cells stably expressing wild type SERT tagged with a yellow fluorescent protein at the N terminus (YFP-human SERT (hSERT)) and incubated in the presence of 150 mm NaCl or of 150 mm choline chloride, conditions known to promote the outward facing and the inward facing conformation, respectively. We monitored the fate of the N terminus by immunoblotting for the YFP moiety, which had been fused to the N-terminal end of SERT. It is evident from Fig. 1 that the N terminus was rapidly cleaved in the presence of choline chloride (Fig. 1, *a*, *lanes labeled ChCl*, and *b*, *second bar*); in contrast, the immunoreactivity of YFP was resistant to tryptic digestion for up to 5 min if the membranes were incubated in the presence of NaCl (Fig. 1, *a*, *lanes labeled NaCl*, and *b*, *third bar*). Importantly, addition of *p*-chloroamphetamine (*p-*CA) also afforded a protection against tryptic removal of the N-terminal YFP tag (Fig. 1, *a*, *lanes labeled ChCl* + *p-CA*, and *b*, *fourth bar*). The combination of NaCl and *p*-chloroamphetamine did not result in any appreciable (*i.e.* statistically significant) additional protection (Fig. 1, *a*, *lanes labeled NaCl* + *p-CA*, and *b*, *fifth bar*). Similar findings were obtained if immunodetection was done with a monoclonal antibody directed against the C terminus of SERT (data not shown). As an additional control, we verified that the proteolytic activity of trypsin was not affected by any of the conditions (if trypsin was allowed to digest albumin in the presence of NaCl or choline chloride with or without *p*-chloroamphetamine) (not shown).

##### Forward and Reverse Transport by N-terminal SERT Truncation Variants

**FIGURE 2.**
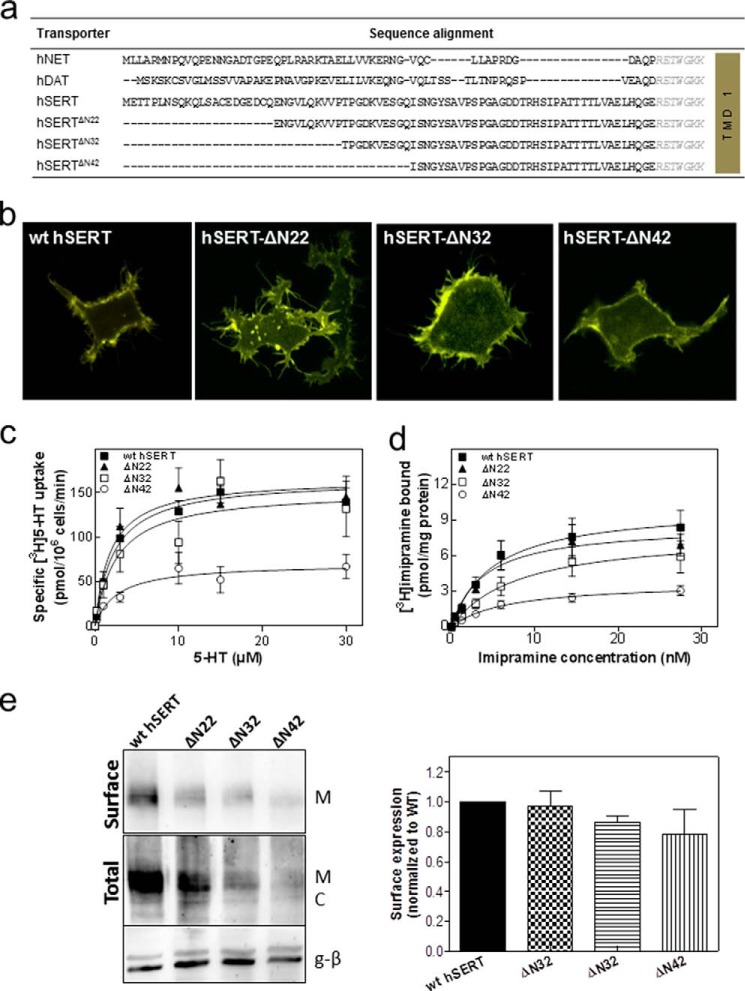
**Alignment of the N-terminal sequences of NET, DAT, and SERT (*a*); cellular distribution (*b*); substrate uptake (*c*); and inhibitor binding of YFP-tagged SERT-ΔN22, SERT-ΔN32, and SERT-ΔN42 in comparison with wild type SERT.** *a*, comparison of the amino acid sequence of the N-terminal region of the human (*h*) monoamine transporter subfamily, indicating poor sequence conservation apart from the highly conserved RETWGKK motif (in *italics*) adjacent to the first transmembrane helix (*TMD 1*). The N terminus of hSERT comprises 85 amino acids. The first 22, 32, or 42 amino acids of the N terminus were truncated, resulting in SERT-ΔN22, SERT-ΔN32, and SERT-ΔN42. *b*, representative images showing the cellular localization of YFP-tagged wild type SERT, SERT-ΔN22, SERT-ΔN32, and SERT-ΔN42 obtained by confocal laser-scanning microscopy. *c*, serotonin uptake by HEK293 cells expressing YFP-tagged wild type SERT (*closed squares*), SERT-ΔN22 (*closed triangles*), SERT-ΔN32 (*open squares*), and SERT-ΔN42 (*open circles*). Cells were incubated with the indicated concentrations of [^3^H]serotonin for 1 min at 22 °C as outlined under “Experimental Procedures.” Nonspecific uptake was determined in the presence of 10 μm paroxetine and subtracted (<10% of total uptake). Data are means ± S.E. from four independent experiments carried out in triplicate. *d*, membranes (15–25 μg/assay) prepared in parallel from the same transiently transfected HEK293 cells shown in *c* were incubated with the indicated concentrations of [^3^H]imipramine for 20 min at 22 °C as outlined under “Experimental Procedures.” Nonspecific binding was determined in the presence of 10 μm paroxetine and subtracted (<20% of total binding at the highest concentration of [^3^H]imipramine). Data are means ± S.E. from four independent experiments carried out in duplicate. The *solid lines* in *c* and *d* were drawn by subjecting the data to a non-linear least square fit to the equation for a rectangular hyperbola. *e*, biotinylation of cell surface proteins was carried out in HEK cells expressing the indicated versions of SERT. *Top panel*, surface expression; *bottom panels*, total lysate fractions and G protein β-subunit (*g-*β) (as a loading control). The integrated intensity of the biotinylated bands (*M*) was quantified by ImageJ relative to the total amount of SERT immunoreactivity in the lysate (*i.e.* the bands with mature glycosylation (*M*) and core glycosylation (*C*)) and normalized to wild type. The data presented in the bar graph are means ± S.E. (*error bars*) (*n* = 4).

If the sequences of the N termini of NET, DAT, and SERT are aligned, it is not possible to identify a any conserved element other than a highly conserved stretch of amino acids (RETWGKK; delineated in *italics* in Fig. 2*a*) preceding the first transmembrane helix (Fig. 2*a*). However, this cannot be important to support amphetamine-induced efflux because it is present in SERT-ΔN64 (truncated amino acids shown in Fig. 2*a*). In this truncated variant, amphetamine-induced reverse transport is severely compromised (20). Accordingly, we created a series of truncation mutants by deleting the first 22, 32, or 42 residues from the SERT N terminus (referred to as SERT-ΔN22, SERT-ΔN32, and SERT-ΔN42, respectively; Fig. 2*a*). We verified that these SERT variants were expressed at the cell surface (Fig. 2*b*). SERT-ΔN22, SERT-ΔN32, and SERT-ΔN42 accumulated their cognate substrate serotonin (Fig. 2*c*) and bound the inhibitor radioligand [^3^H]imipramine (Fig. 2*d*) with an affinity comparable with that of wild type SERT (Table 1). It is also evident from Fid. 2*c* that the maximum transport rate (*V*_max_) catalyzed by SERT-ΔN42 was lower than that observed with the other SERT variants. However, this was also true for the *B*_max_ of inhibitor binding. Thus, if the *V*_max_ of substrate uptake was related to the *B*_max_ of radioligand binding, the estimated turnover number of SERT-ΔN42 was not reduced; in fact, the turnover number of all truncation mutants was reasonably comparable with that of wild type SERT (Table 1). Hence, we concluded that the reduced *V*_max_ and *B*_max_ of SERT-ΔN42 were linked to lower expression levels as shown by cell surface biotinylation experiments (Fig. 2*e*).

**TABLE 1:** **Kinetic parameters determined from radiolabeled serotonin uptake and imipramine binding studies** HEK293 cells were transiently transfected with the plasmids encoding wild type hSERT or the N-terminal truncations thereof. After 48 h, specific 5-[^3^H]HT uptake was measured. [^3^H]Imipramine binding assays were carried out in membranes preparations (see “Experimental Procedures”). *K_m_*, *V*_max_, *K_D_*, and *B*_max_ values were determined from data shown in Fig. 2, *C* and *D. K_i_* values of *p*-CA or ibogaine were calculated from inhibition of [^3^H]imipramine binding data shown in Fig. 3, *C* and *D*. The values are represented as arithmetic means ± S.E. from at least two independent experiments performed in duplicate (binding) or triplicate (uptake). Different conditions were statistically compared by one-factor analysis of variance followed by Tukey's post hoc *t* tests.

Transporter	*V*_max_	*K_m_*	*B*_max_	*K_D_*	Turnover number	*K_i_ p*-CA	*K_i_* ibogaine
	*pmol*/*10^6^cells min*^−*1*^	μ*m*	*pmol*/*mg protein*	*nm*	*min*^−1^	μ*m*	μ*m*
SERT-WT	182.1 ± 35.0	2.3 ± 0.6	10.1 ± 1.4	4.9 ± 2.0	186	1.9 ± 0.2	2.8 ± 0.5
SERT-ΔN22	181.3 ± 24.6	1.8 ± 0.7	8.6 ± 1.0	3.8 ± 1.4	195	2.2 ± 0.5	2.8 ± 0.8
SERT-ΔN32	203.4 ± 11.3	2.6 ± 1.5	8.0 ± 1.6	7.9 ± 4.0	239	2.3 ± 0.4	5.3 ± 1.3
SERT-ΔN42	86.8 ± 26.7*^a^*	2.6 ± 1.2	3.7 ± 0.5*^a^*	6.6 ± 2.3	257	2.9 ± 2.0	5.4 ± 0.6

*^a^ p* < 0.001, significantly different from control.

**FIGURE 3.**
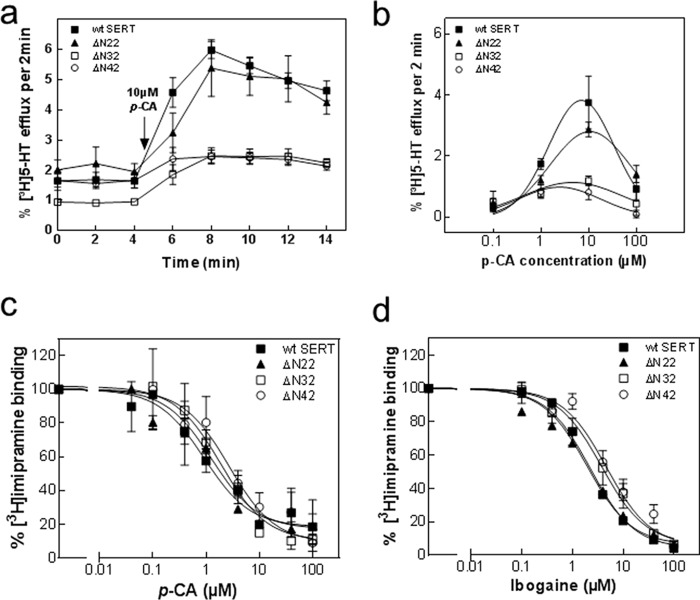
***p-*Chloroamphetamine-induced efflux of [^3^H]serotonin from HEK293 cells expressing wild type SERT, SERT-ΔN22, SERT-ΔN32, and SERT-ΔN42 (*a* and *b*) and inhibition of [^3^H]imipramine binding to membranes from these cells by *p-*chloroamphetamine (*c*) and ibogaine (*d*).** *a*, HEK293 cells transiently expressing either YFP-tagged wild type SERT (*closed squares*), SERT-ΔN22 (*closed triangles*), SERT-ΔN32 (*open squares*), and SERT-ΔN42 (*open circles*) were preloaded with 0.4 μm [^3^H]serotonin for 20 min at 37 °C and subsequently superfused with Krebs-HEPES buffer for 45 min at 22 °C to obtain a stable basal efflux as outlined under “Experimental Procedures.” Thereafter, three fractions (2 min each) were collected to verify stable efflux. Subsequently, efflux was triggered by superfusion of the cells with 10 μm *p*-CA (administration indicated in the graph by an *arrow*), and five fractions (2 min each) were collected. Data are expressed as fractional release (see “Experimental Procedures”) and represent means ± S.E. from four independent experiments carried out in triplicate. *b*, concentration-response curve for *p*-chloroamphetamine-triggered efflux of [^3^H]serotonin. The experiments were done as in *a* in the presence of the indicated concentrations of *p*-chloroamphetamine. Plotted is the peak efflux from which basal efflux was subtracted. Data are means ± S.E. from four independent experiments carried out in triplicate. *c* and *d*, membranes (15–25 μg/assay) prepared from HEK293 cells expressing YFP-tagged wild type SERT (*closed squares*), SERT-ΔN22 (*closed triangles*), SERT-ΔN32 (*open squares*), and SERT-ΔN42 (*open circles*) were incubated in the presence of 5 nm [^3^H]imipramine and the indicated concentrations of *p*-chloroamphetamine (*c*) and ibogaine (*d*) for 20 min at 25 °C. Nonspecific binding was determined in the presence of 10 μm paroxetine and subtracted to obtain specific binding. In the absence of competitors, specific binding was in the range of 30 and 100 fmol and defined as the 100% reference value to normalize for interassay variations. Data are means ± S.E. from three to four independent experiments carried out in duplicate. *Error bars* represent S.E.

Major differences were however apparent if HEK293 cells expressing SERT-ΔN22, SERT-ΔN32, or SERT-ΔN42 were preloaded with [^3^H]serotonin and subsequently superfused with *p*-chloroamphetamine: cells expressing SERT-ΔN32 and SERT-ΔN42 released only very low amounts of [^3^H]serotonin when challenged with 10 μm *p*-chloroamphetamine (Fig. 3*a*, *open symbols*). In contrast, *p*-chloroamphetamine-triggered substrate efflux through SERT-ΔN22 and wild type SERT was comparable (Fig. 3*a*, *closed symbols*). The reduced efficacy of *p*-chloroamphetamine was not remedied by raising its concentration: in fact, a bell-shaped concentration response curve was seen with all variants of SERT (Fig. 3*b*), which is expected because at high concentrations amphetamines occupy all SERT moieties and renders them unavailable for serotonin efflux (10, 20). We also independently verified that all three mutants bound *p*-chloroamphetamine with an affinity similar to that of wild type SERT by assessing the ability of *p*-chloroamphetamine to displace [^3^H]imipramine (Fig. 3*c*). Amphetamine-induced substrate efflux is reduced by mutations, which increase the dwell time of the resulting transporter variants in the inward facing conformation (20). Ibogaine traps SERT (and other monoamine transporters) in the inward facing conformation (21, 22). Accordingly, transporter mutants in which the conformational equilibrium is shifted to the inward facing state display higher affinity for ibogaine (20). We therefore used ibogaine to examine whether the N-terminal truncation increased the dwell time of the transport in the inward facing conformation. It is evident from Fig. 3*d* and Table 1 that truncation of the N terminus by up to 42 amino acids did not affect the affinity of the resulting SERT variants for ibogaine.

##### Analysis of Role of the N Terminus in the Transport Cycle of SERT

**FIGURE 4.**
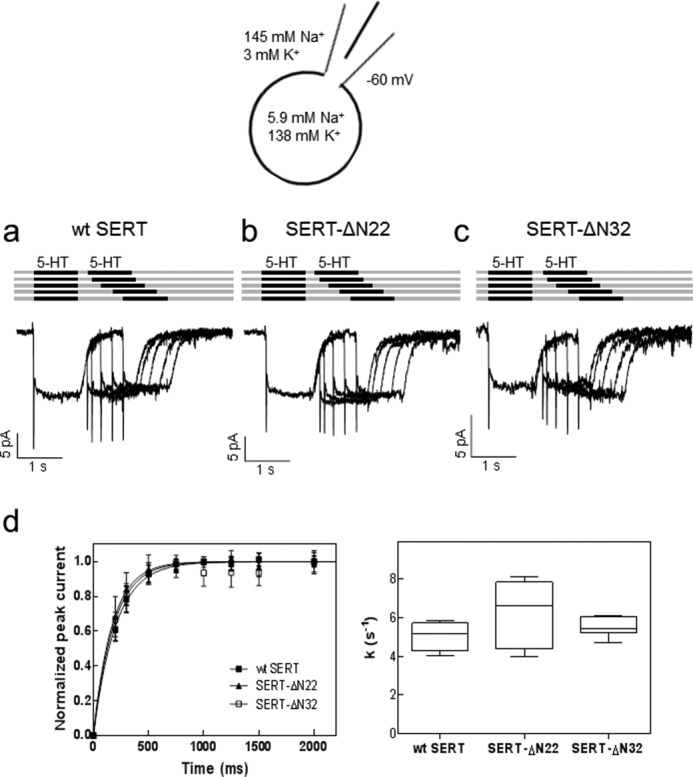
**Substrate-induced currents elicited by paired pulse applications of serotonin to HEK293 cells expressing wild type SERT (*a*), SERT-ΔN22 (*b*), and SERT-ΔN32 (*c*) in the presence of a physiological Na^+^ gradient.** *a–c*, peak current recovery protocol and representative traces. 5-HT (10 μm) was applied for 1 s followed by variable (increasing) washout intervals and subsequent 5-HT test pulses. The current amplitude of the peak current elicited by 5-HT represents the fraction of transporters available for 5-HT binding. Under the ionic conditions used (see schematic rendering), the transporter operates in the forward transport mode, which is evident from the steady state component of the current. Accordingly, the size of the second peak current reflects the fraction of SERT that returned to the outward facing conformation. *d*, the time course of peak current recovery was plotted for wild type SERT (*closed squares*), SERT-ΔN22 (*closed triangles*), and SERT-ΔN32 (*open squares*) and fitted to a monoexponential function. Data are means ± S.D. (*error bars*) from six independent experiments. The box plot shows the median recovery rates (*k*) and the interquartile range extracted from the individual monoexponential recovery curves; *whiskers* show maximum and minimum values. A Kruskal-Wallis test did not reveal any significant differences between intergroup and intragroup variation (*p* = 0.39).

Substrate uptake and release assays only provide limited information on the transport cycle of SERT. The comparison of the maximum transport velocity (*V*_max_) and SERT density (*B*_max_) assessed by radioligand binding allows estimation of the turnover number. However, this number is imprecise because it is obtained as a ratio; thus it is subject to uncertainty due to error propagation arising from the division of two large numbers. However, the transport cycle of monoamine transporters is accessible with high time resolution if substrate-induced currents through SERT are recorded in the patch clamp mode (2, 23). Two current components can be readily distinguished (Fig. 4*a*). (i) Rapid application of substrate elicits a peak current, which is carried by the valence of the translocated sodium ion (4). Accordingly, the peak current monitors the conformational transition from the outward facing to the inward facing state. (ii) The peak current is followed by a sustained current, which persists as long as the cell is superfused with substrate. This current arises from a conducting state, which is visited during the return step, *i.e.* when the K^+^-bound (or H^+^-bound) SERT switches back from the inward facing state to the outward facing state, which is the rate-limiting step in the transport cycle (2, 4). Accordingly, it is possible to monitor the progression through the transport cycle by resorting to a paired pulse protocol in the presence of a physiological ion gradient (*i.e.* high extracellular Na^+^ and low extracellular K^+^/low intracellular Na^+^ and high intracellular K^+^); the first pulse of serotonin triggers the transport cycle. Because the return step is rate-limiting (2, 3), SERT accumulates in the inward facing state. Thus, a prolonged washout period is required until all transporter molecules have undergone the conformational transition to the outward facing state such that a second pulse of substrate gives rise to a full-fledged peak current. We compared the recovery rate of wild type SERT (Fig. 4*a*), SERT-ΔN22 (Fig. 4*b*), and SERT-ΔN32 (Fig. 4*c*). The recovery was adequately described by a monoexponential rise to a maximum in all instances (Fig. 4*d*), and the calculated rate constants were comparable (Fig. 4*d*). Hence, this comparison showed that these three SERT variants operated with a similar rate in the forward transport mode.

**FIGURE 5.**
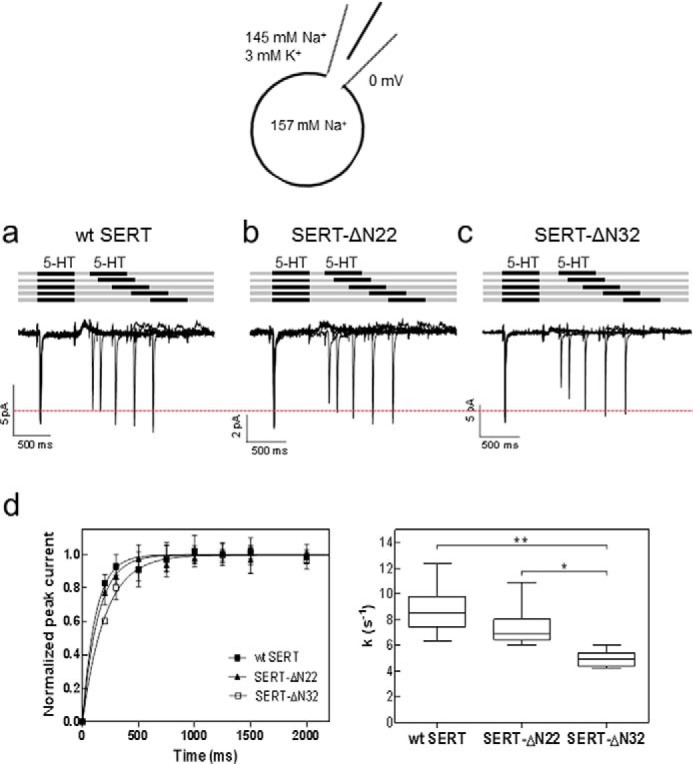
**Substrate-induced peak currents elicited by paired pulse applications of serotonin to HEK293 cells expressing wild type SERT (*a*), SERT-ΔN22 (*b*), and SERT-ΔN32 (*c*) in the presence of high internal Na^+^.** *a–c*, peak current recovery protocol and representative traces. 5-HT (10 μm) was applied for 0.5 s followed by variable (increasing) washout intervals and subsequent 5-HT test pulses. The presence of high internal Na^+^ precludes progression through the transport cycle, thus resulting in the suppression of the steady state current (Fig. 4, *a–c*) and forces SERT into the exchange mode. The recovery of the peak current (elicited by the second 5-HT pulse) therefore monitors the fraction of transporters that have completed the half-cycle to return to the outward facing conformation and are available for renewed binding of serotonin. *d*, the time course of peak current recovery was plotted for wild type SERT (*closed squares*), SERT-ΔN22 (*closed triangles*), and SERT-ΔN32 (*open squares*) and fitted to a monoexponential function. Data are means ± S.D. (*error bars*) from six independent experiments. The box plot shows the median recovery rates (*k*) and the interquartile range extracted from the individual monoexponential recovery curves; *whiskers* show maximum and minimum values. The recovery rate for SERT-ΔN32 was significantly lower (Kruskal-Wallis test followed by Dunn's multiple comparisons; *, *p* < 0.05; **, *p* < 0.01).

In the presence of high internal sodium concentrations and in the absence of internal K^+^, release of Na^+^ from the inward facing conformation is precluded, and the transporter cannot progress through the transport cycle (2, 4, 24). Accordingly, the steady state component of the current is eliminated because the substrate-free return step is eliminated and the transporter can only seesaw through half-cycles (Figs. 5*a* and 4*a*). Thus, recording the isolated peak current allows for monitoring the exchange mode of the transporter because the transporter can only return from the inward facing state to the outward facing conformation in the Na^+^- and substrate-bound form (25). This return step can again be monitored by a protocol of paired substrate pulses, which allows for detection of the time interval required to recover a full-fledged isolated peak current (Fig. 5*a*) and thus the ability of SERT to operate in the exchange mode. The peak current recovered within comparable time ranges in wild type SERT (Fig. 5*a*) and SERT-ΔN22 (Fig. 5*b*). In contrast, a delay was observed with SERT-ΔN32 (Fig. 5*c*). The difference between wild type SERT and SERT-ΔN22 on the one hand and SERT-ΔN32 on the other hand can be readily appreciated if the peaks elicited by the first paired pulse are compared (Fig. 5, *a–c*, highlighted by the *red dotted line*). The delayed peak current recovery of SERT-ΔN32 is also evident from the time course plotted in Fig. 5*d*. We calculated the rate constants by fitting the data to the equation for a monoexponential rise to a maximum to verify that the rate constant observed with SERT-ΔN32 was significantly smaller than that of wild type SERT and SERT-SERT-ΔN22 (Fig. 5*d*). In fact, the rate constant of wild type SERT was about 2-fold larger than that of SERT-ΔN32. Thus, SERT-ΔN32 needed twice as long as wild type SERT to operate in the reverse mode. The rate of entry of SERT-ΔN32 into the reverse mode (5.0 ± 0.6 s^−1^; Fig. 5*d*) was of the same magnitude as the rate of the return step (5.5 ± 0.5 s^−1^; Fig. 4*d*), which is the rate-limiting step in the forward cycle mode. However, we stress that a comparison of these two rates is not meaningful because they are obtained under vastly different ionic conditions.

## Discussion

Molecular dynamics simulations predict that the N terminus of DAT is flexible, engages PIP_2_, triggers the opening of the inner gate of via an interaction with intracellular loop 4, and thus promotes release of Na^+^ from the Na2 site; these actions are causally related because the N terminus is directed to IL-4 by engaging PIP_2_ molecules (18). Amphetamine-induced reverse transport by SERT is also dependent on PIP_2_ (17). It is therefore safe to assume that the N terminus of SERT operates in a manner analogous to that of DAT, although their N-terminal sequences are divergent. The current findings are consistent with several predictions of the original lever hypothesis (20). (i) Our experiments showed that the conformation of the N terminus differed in the inward and the outward facing conformation and, most importantly, that binding of *p*-chloroamphetamine to SERT rendered the N terminus partially resistant to tryptic cleavage. This unequivocally proves that the signal arising from occupancy of the binding site by an amphetamine is relayed from the hydrophobic core to the N terminus. (ii) The N terminus is dispensable for the forward transport mode of SERT. This conclusion is based on the analysis of both the transport rate and the turnover number by a combination of uptake and binding assays and an analysis of the transport cycle by electrophysiological recordings. Moreover, Sweeney *et al.* (26) have very recently used a chimeric approach to identify a role for the N terminus of DAT, but not of SERT, in specifying the affinity of DAT for substrates and several inhibitors. (iii) In contrast, truncation of the N terminus by ≥32 residues greatly reduced the propensity of the transporter to enter the reverse mode. This conclusion is again based on two independent lines of evidence, *i.e.* the reduced amphetamine-induced efflux of serotonin from cells expressing SERT-ΔN32 (and SERT-ΔN42), which was observed in superfusion experiments, and the delayed entry of SERT-ΔN32 into the reverse transport mode, which was verified in patch recordings under conditions that precluded progression through the transport cycle. It is therefore reasonable to posit that the amphetamine-induced change in the conformation of the N terminus is causally linked to the requirement of the N terminus in amphetamine-induced reverse transport.

**FIGURE 6.**
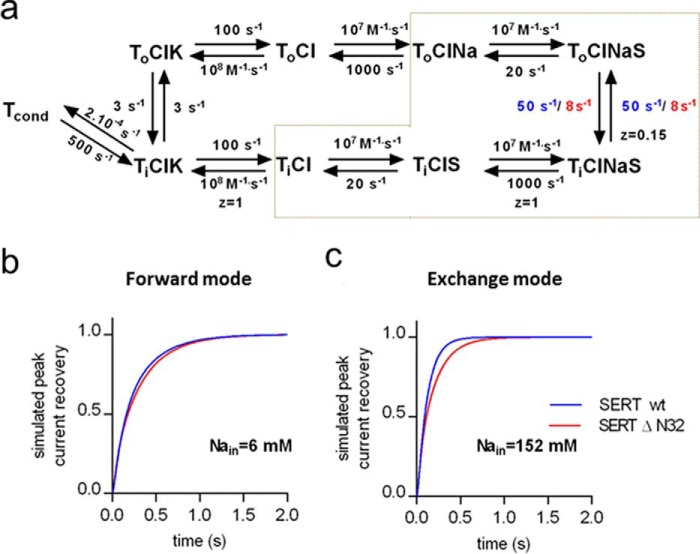
**A concise kinetic model of the transport cycle of SERT.** *a*, schematic representation of the transport cycle. *T_o_* and *T_i_* refer to the outward and inward facing conformations of the transporter, respectively. T_o_ sequentially binds sodium (*Na*) and substrate (*S*) in the outward facing state, switching to the inward facing state where it releases sodium and substrate and takes up potassium (*K*) to return to the outward facing state and thus complete the cycle. T_cond_, conducting state of the transporter. Rate constants for the individual steps are placed adjacent to the corresponding reaction *arrows*. The association and dissociation of chloride was omitted to simplify the kinetic modeling. *z* indicates the valence of the pertinent reaction. In the exchange mode, the transporter only visits the conformational states encompassed by the *gray dashed lines*. The time course of peak current recovery of wild type SERT and SERT-Δ32N was simulated by allowing the indicated rate constants (wild type SERT in *blue* and SERT-Δ32N in *red*) to vary. All other parameters were shared in the simulations, and the concentrations of Na^+^, K^+^, and substrate were set to the concentrations used in the experiments. *b*, time course of peak current recovery simulated for the recording condition of Fig. 4 in which the transporter favors the forward mode. *c*, time course of peak current recovery for the condition of Fig. 5 in which the transporter favors the exchange mode. The solution in which the rate of transition between inward and outward facing step differs by a factor of 6 recapitulates the experimental observation (Figs. 4*d* and 5*d*).

The conformations of the N and C termini are not accessible from the crystal structures of SERT (27) and DAT (25). To the best of our knowledge, our findings provide the first direct experimental evidence for a change in the conformation of the N terminus when SERT switches from the outward facing conformation to the inward facing conformation: the N terminus was protected against tryptic digestion in the presence of Na^+^, but it was highly susceptible to cleavage in the presence of equimolar choline; these ionic conditions favor the outward and inward facing conformations, respectively (28). Computational studies conclude that the N terminus of DAT is highly mobile (18). The relation of these molecular dynamics simulations to our findings, however, is not clear for several reasons. Our experiments did not allow determination of whether the conformation of the N terminus differed in the fully loaded transporter (*i.e.* liganded with two Na^+^, one Cl^−^, and one substrate molecule) from that in the co-substrate-bound state (*i.e.* with two Na^+^ and one Cl^−^ bound) because the sole addition of NaCl sufficed to afford substantial protection, which was not further augmented to an appreciable extent by amphetamines (or serotonin; not shown). In contrast, in the absence of Na^+^, binding of *p*-chloroamphetamine afforded protection of the N terminus. However, this species was not investigated in the computational studies of DAT (18). In addition, there are four arguments that question a specific role of the N terminus in the forward transport mode of SERT. (i) The N terminus of SERT can be truncated (by up to 64 amino acids; see Ref. 20), and uptake is not impaired. (ii) This was recapitulated in the present experiments where we examined the transport cycle in real time: SERT-ΔN32, for instance, cycled in the forward mode with a rate constant that was indistinguishable from that of wild type SERT, but it was greatly impaired in its ability to operate in the substrate exchange mode. (iii) PIP_2_ is required for entry of SERT into the substrate exchange because PIP_2_ depletion suppresses amphetamine-induced efflux through SERT. However, PIP_2_ depletion does not affect substrate uptake, *i.e.* the forward transport mode (17). (iv) The N terminus of DAT can be tethered by introducing an additional N-terminal transmembrane helix. This transporter can operate in the forward transport mode to support concentrative influx of dopamine, but amphetamine-induced dopamine efflux is severely impaired (20). This indicates that the flexibility of the N terminus is more important for supporting the exchange mode. Dissociation of Na^+^ (and of substrate) followed by binding of K^+^ (or of H^+^) to the inward facing conformation specifies the forward transport mode (4): accordingly, raising intracellular Na^+^ *a priori* increases the propensity of SERT to switch into the substrate exchange mode. This can also be appreciated from the reaction scheme of the transporter cycle shown in Fig. 6*a*. In the exchange mode, the transporter seesaws through the half-cycle delineated by the *gray dashed lines* in Fig. 6*a*. The scheme also delineates a simplified version of the kinetic model proposed by Hasenhuetl *et al.* (4). In our calculations, we eliminated two reactions that are immaterial to the comparison of wild type SERT and SERT-ΔN32, *i.e.* (i) the effect H^+^ binding on the return step (which is only seen at pH ≪ 7) and (ii) the association and dissociation of Cl^−^, which was omitted. We used this kinetic model to estimate the effect resulting from the N-terminal truncation. The rate constants for the individual reactions other than the transition from the outward facing (*T_o_ClNaS*) to the inward facing substrate- and co-substrate-loaded transporter (*T_i_ClNaS*) were assumed to be identical for wild type SERT and SERT-ΔN32. Under these conditions, the kinetic model is capable of simulating the results from both the forward cycle mode (Figs. 6*b* and 4*d*) and the exchange mode (Figs. 6*c* and 5*d*) if the rate constants for the transition step differ by a factor of ∼6 (*i.e.* 50 and 8 s^−1^ for wild type SERT and SERT-ΔN32, respectively).

Our observations are consistent with a model where the N terminus cooperates with the ion exchange reaction to determine the set point where the switch to the exchange mode occurs. This allows the N terminus to integrate additional signals arising *e.g.* from phosphorylation (8–15) or from the recruitment of additional interaction partners, *e.g.* syntaxin 1A (19) or PIP_2_ (16, 17). It is worth pointing out that in the computational studies of the DAT N terminus opening of the inner vestibule to allow for Na^+^ release was contingent on the presence of PIP_2_ in the simulated lipid bilayer. In the absence of PIP_2_, the N terminus of DAT was not directed to intracellular loop 4, and the ionic locks that sealed the inner gate were not opened such that an exit pathway for Na^+^ and substrate release failed to form (18). Based on our observations and based on the arguments listed above, we propose that the computational exercise of Khelashvili *et al.* (18) captured several important mechanistic aspects that are relevant to understand the role of the N terminus in triggering amphetamine-induced exchange mode but that may not be necessary in the forward transport mode (where the N terminus is dispensable).

The lever hypothesis predicts that upon binding of amphetamines to one SERT moiety within the SERT oligomer the N terminus undergoes a conformational change to promote serotonin release through a different moiety (20). This model is supported by circumstantial evidence (10, 17, 20) but also inspired by the analogy with BetP. In this bacterial betaine transporter, the communication between individual moieties in the trimer is accomplished by the C terminus, which is topologically equivalent to the N terminus (30). BetP responds to osmotic stress by facilitating cellular uptake of betaine. This response is contingent on oligomer formation (31, 32) and involves a K^+^-sensor: acidic lipids (phosphatidylglycerols) are bound to BetP and engage the C terminus. Osmotic stress results in an increase in the intracellular concentration of K^+^, which releases the C terminus by obviating its interaction with the phospholipid headgroups (33) and thus drives formation of salt bridge between the C terminus and intracellular loop 2. The signal is transmitted to the extracellular side by helix 3 and results in the formation of a vestibular binding site for betaine (34). There is another example where regulation of transport activity relies on the oligomeric arrangement: in the ammonium transporters AMT1;1 and AMT1;3 of *Arabidopsis thaliana*, the C-terminal domain of one moiety transactivates the adjacent transporter in the trimer; this allows for integration of inhibitory input because phosphorylation of Thr-460 and of Thr-472 in AMT1;1 and AMT1;3, respectively, eliminates the stimulation (35) analogously to the postulated mechanism. Our experiments were not designed to examine whether the N terminus of SERT acted in *cis* or in *trans*, *i.e.* whether it engaged a segment of the moiety from which it originated or a neighboring transporter molecule to trigger the exchange mode. However, the truncation mutations do provide a means to search for biochemical complementation. The prediction is that a transport cycle-deficient mutant ought to restore amphetamine-induced efflux to SERT-ΔN32 and SERT-ΔN42 provided that it can bind amphetamine and supply the N terminus for the intermolecular interaction in *trans*. This is a testable prediction.

## Experimental Procedures

### 

#### 

##### Materials

[^3^H]Imipramine (40 Ci/mmol) and [^3^H]serotonin (28 Ci/mmol) were obtained from PerkinElmer Life Sciences. The QuikChange^TM^ II site-directed mutagenesis kit was from Agilent Technology, Inc. (Santa Clara, CA). Oligonucleotides were purchased from Operon Biotechnologies (Cologne, Germany). The polyclonal rabbit antibody against GFP/YFP (ab290) was from Abcam plc (Cambridge, UK). The generation of the monoclonal mouse antibody against the C terminus of SERT has been described (36). ECL^TM^ anti-rabbit IgG (from donkey) and anti-mouse (from sheep) secondary antibodies linked to horseradish peroxidase for primary antibody detection were from GE Healthcare. Ibogaine was a generous gift of Sacrament of Transition (Maribor, Slovenia). *p*-Chloroamphetamine, paroxetine, cocaine, buffers, and salts were from Sigma-Aldrich. Cell culture media, supplements, and antibiotics were obtained from Invitrogen. Sequencing grade modified trypsin and soybean trypsin inhibitor were purchased from Promega (Madison, WI) and from Sigma-Aldrich, respectively. Bovine serum albumin (BSA) and Complete protease inhibitor mixture tablets were from Roche Applied Science. Scintillation mixture for radioactive measurement (Rotiszint® Eco plus) was from Carl Roth GmbH and Co.

##### Plasmid Construction

The truncation mutants ^ΔN22^SERT-YFP, ^ΔN32^SERT-YFP, and ^ΔN42^SERT-YFP were generated using the cDNA encoding human wild type SERT as template (cloned into the pEYFP vector (Clontech)) using the QuikChange II site-directed mutagenesis kit with the following primers (sequences shown are the sense strand in the 5′ to 3′ direction): SERT-ΔN22, 5′-GCGCAAGCTTATGGAAAACGGAGTTCTACAG-3′; SERT-ΔN32, 5′-GCGCAAGCTTATGACCCCAGGGGACAAAG-3′; SERT-ΔN42, 5′-GCGCAAGCTTATGTCCAATGGGTACTCAGC-3′. All mutations were confirmed by sequencing.

##### Cell Culture and Transfection

HEK293 cells were cultured in Dulbecco's modified Eagle's medium (DMEM) with high glucose (4.5 g/liter) and l-glutamine (584 mg/liter) supplemented with 10% fetal calf serum (FCS) and a mixture of penicillin/streptomycin (50 mg/liter) at 37 °C in a 5% CO_2_ humidified atmosphere. Cells were seeded onto poly-d-lysine-coated 48-well culture plates (for uptake assays), 15-cm dishes (for cell membrane preparations), 15-mm glass coverslips (for confocal microscopy experiments), 5-mm glass coverslips (for release assays), or 3-cm dishes (for electrophysiological recordings). Transient transfections were done using the calcium phosphate precipitation method with 3.4–40 μg of cDNA. In some instances, stably transfected HEK293 cells were created by using geneticin (G418) for selection.

##### Laser-scanning Confocal Microscopy

Confocal microscopy was carried out as described earlier (28). HEK293 cells (3 × 10^5^) were seeded onto PDL-coated 15-mm coverslips and transfected with plasmids encoding YFP-tagged wild type and mutant versions of SERT after 24 h using calcium phosphate-mediated precipitation. After an additional 48-h interval, images of the cells (maintained in Krebs-HEPES buffer containing 10 mm HEPES, 120 mm NaCl, 3 mm KCl, 2 mm CaCl_2_, 2 mm MgCl_2_, and 2 mm glucose monohydrate, pH adjusted to 7.4 with NaOH) were captured with a Zeiss Axiovert LSM510 confocal laser-scanning microscope (argon laser, 30 milliwatts; helium/neon laser, 1 milliwatt; equipped with an oil immersion objective (Zeiss Plan-NeoFluar ×40/1.3)). The images were analyzed with Zeiss LSM Image Browser (version 4.2.2.121; Carl Zeiss Microimaging GmbH, Oberkochen, Germany).

##### Membrane Preparation and Radioligand Binding

Membranes were prepared from transiently and stable transfected HEK293 cells as outlined (28). For saturation binding experiments, the HEK293 cell membranes (15–25 μg/assay) were incubated for 20 min at 22 °C with [^3^H]imipramine (concentrations ranging from 0.2 to 28 nm) in buffer (final volume, 0.2 ml) containing 20 mm Tris-HCl, pH 7.5, 1 mm EDTA, 2 mm MgCl_2_, 120 mm NaCl, and 3 mm KCl). Nonspecific binding was defined in the presence of 10 μm paroxetine. Competition binding experiments were done under the same conditions in the presence of 5 nm [^3^H]imipramine and logarithmically spaced concentrations of *p-*chloroamphetamine (ranging from 0.1 to 100 μm) or of ibogaine (ranging from 0.03 to 100 μm). The binding reaction was terminated by rapid filtration over glass fiber filters (Whatman GF/B), which had been presoaked with 0.5% polyethyleneimine. Filters were washed with buffer (20 mm Tris-HCl, pH 7.5, 2 mm MgCl_2_, and 120 mm NaCl), transferred into counting vials, and overlaid with scintillation fluid. The radioactivity trapped on the filter was quantified by liquid scintillation counting at 50% efficiency.

##### Tryptic Digestion, Cell Surface Biotinylation, and Immunoblotting

The membrane suspension (20 μg/assay; prepared from HEK293 cells stable expressing wild type SERT) was incubated with trypsin (1.6 μg) at 22 °C in a final volume of 10 μl containing buffer (20 mm HEPES·NaOH, pH 7.4, 1 mm EDTA, 2 mm MgCl_2_, and 150 mm NaCl, or 150 mm choline chloride) and where indicated 10 μm *p*-chloroamphetamine. The reaction was stopped after 1 or 5 min by the addition of soybean trypsin inhibitor (8 μg). Thereafter, the sample was dissolved in sample buffer containing 20 mm dithiothreitol and 1% SDS. Aliquots corresponding to 50% of the reaction mixture were subjected to denaturing polyacrylamide gel electrophoresis; the resolved proteins were transferred onto nitrocellulose membranes, which were probed with either a rabbit antibody directed against GFP (diluted 1:5000 in Tris-buffered saline containing 20 mm Tris-HCl, pH 7.5, 150 mm NaCl, 0.1% Tween, and 1% BSA) or a mouse monoclonal antibody directed against the C terminus of SERT (diluted 1:500). The immunoreactive bands were detected by enhanced chemoluminescence using horseradish peroxidase-conjugated secondary antibodies and SuperSignal® West Femto Maximal Sensitivity Substrate (Thermo Fisher Scientific). The next day membranes were washed extensively and probed with secondary HRP-linked anti-rabbit antibody against GFP/YFP (diluted 1:8000 in 0.1% TBST with 3% BSA) or anti-mouse antibody against SERT-antibody (diluted 1:2000 in 0.1% TBST with 3% BSA) for 1 h at room temperature. After a second extensive wash with 0.1% TBST, membranes were developed using chemiluminescent substrate SuperSignal West Femto Maximal Sensitivity Substrate.

Biotinylation of cell surface proteins was done as described previously (37). Briefly, HEK cells were incubated in the presence of sulfo-NHS-SS-biotin (sulfosuccinimidyl-2-(biotinamido)ethyl-1,3-dithiopropionate; Pierce; 1 mg/ml) for 30 min. Following lysis of cells, the labeled surface proteins were collected by binding to streptavidin beads (Thermo Fischer Scientific), and the amount of biotinylated SERT was detected by immunoblotting. Band intensities were directly quantified from the photon emission captured by an Alpha Innotech FluorChem® FC2 imager, and integrated density values were calculated with FluorChem software.

##### Serotonin Uptake

Uptake and release of [^3^H]serotonin by HEK293 cells expressing wild type or N-terminally truncated versions of SERT were determined as described previously (20). Briefly, for uptake experiments, the HEK293 cells were seeded onto poly-d-lysine-coated 48-well plates (5 × 10^4^/well). After 16 h, the medium was removed followed by a gentle wash with 1 ml of Krebs-HEPES buffer (10 mm HEPES·NaOH, pH 7.4, 120 mm NaCl, 3 mm KCl, 2 mm CaCl_2_, and 2 mm MgCl_2_ supplemented with 5 mm d-glucose) at 22 °C. Thereafter, the cells were incubated with [^3^H]serotonin (concentration range from 0.2 to 30 μm, progressively diluted with unlabeled serotonin) for 1 min. Subsequently, the medium was removed, and the cells were rapidly rinsed with ice-cold Krebs-HEPES buffer and lysed in 1% SDS. The accumulated radioactivity was quantified by liquid scintillation counting. Nonspecific uptake was determined in the presence of 10 μm paroxetine. For amphetamine-induced efflux of [^3^H]serotonin, HEK293 cells (4 × 10^5^ cells/well) expressing wild type or truncated versions of SERT were seeded onto PDL-coated 5-mm coverslips in 96-well plates. After 24 h, the medium was aspirated, and the cells were preincubated with 0.4 μm [^3^H]serotonin for 20 min at 37 °C in a final volume of 0.1 ml of Krebs-HEPES buffer. The coverslips with the preloaded cells were transferred into a superfusion chamber and superfused with Kreps-HEPES buffer at a flow rate of 0.7 ml/min. A stable baseline in efflux of [^3^H]serotonin was established after 45 min at 25 °C. Thereafter, three fractions (2 min each) were collected to quantify baseline efflux. Subsequently, the cells were superfused with Krebs-HEPES buffer containing 10 μm *p*-chloroamphetamine for 10 min, resulting in five fractions (2 min each), which allowed for quantification of *p*-chloroamphetamine-triggered [^3^H]serotonin efflux. Thereafter, the cells were lysed with 1% SDS to determine the residual amount of [^3^H]serotonin by liquid scintillation counting. The efflux of [^3^H]serotonin is expressed as fractional release, *i.e.* as percentage of the total [^3^H]serotonin present within the cells at the beginning of any given fraction (20).

##### Electrophysiological Recordings

Serotonin-induced currents were recorded in the whole cell patch clamp configuration as described previously (2). Briefly, HEK293 cells were seeded on 3.5-cm dishes (5 × 10^5^ cells/dish) and transiently transfected with either wild type or selected SERT constructs (all GFP-tagged at the N terminus) using Effectene® (Qiagen) or JetPrime® (Polyplus Transfection) according to the manufacturers' protocols. After 24 h, the cells were reseeded onto PDL-coated 3.5-cm dishes at low density. Electrophysiological recordings were done after an additional interval of 24 h. For recordings of the forward transport mode, glass pipettes were filled with an internal solution containing 133 mm potassium gluconate, 5.9 mm NaCl, 1 mm CaCl_2_, 0.7 mm MgCl_2_, 10 mm EGTA, and 10 mm HEPES, pH of 7.2 with KOH. For recording of the substrate-induced peak currents, the internal solution contained 152 mm NaCl, 1 mm CaCl_2_, 0.7 mm MgCl_2_, 10 mm EGTA, and 10 mm HEPES, pH adjusted to 7.2 with NaOH. Cells were continuously superfused at room temperature with an external solution consisting of 140 mm NaCl, 3 mm KCl, 2.5 mm CaCl_2_, 2 mm MgCl_2_, 20 mm glucose, and 10 mm HEPES, pH adjusted to 7.4 with NaOH, by using the Octaflow II system. This system guarantees a complete liquid exchange around the cell environment within ∼100 ms at a constant flow rate. Substrate-induced currents were recorded using an Axopatch 200B amplifier and pClamp 10.2 software (MDS Analytical Technologies, Sunnyvale, CA). Cells were voltage-clamped at a holding potential of −60 or 0 mV for peak current recordings done with low and high internal Na^+^, respectively. Serotonin (10 μm) was applied for 1 or 0.5 s for recordings done in the presence of low and high internal Na^+^ concentrations, respectively. Peak current recovery was recorded by first applying 10 μm serotonin (resulting in a reference peak) and subsequently superfusing external solution for increasing time intervals followed by reapplying 10 μm serotonin (resulting in a second peak current, the magnitude of which progressively increased over time). The intervals between first and second serotonin application lasted for 200, 300, 500, 750, 1000, 1250, 1500, 2000, 3000, and 5000 ms. Current traces were filtered at 1 kHz and digitized with a Digidata 1550 (MDS Analytical Technologies). The currents were quantified using Clampfit 10.2 software. Passive holding currents were subtracted, and traces were filtered by a 100-Hz digital Gaussian low pass filter. Peak current recovery was assessed by calculating the ratio of the amplitudes of the recovered peak current at any given time interval over the reference peak current triggered by the first application of serotonin. The kinetic modeling was done as described by Hasenhuetl *et al.* (4).

##### Statistics

If not indicated otherwise, data are expressed as arithmetic means ± S.E. Data points obtained in binding and uptake experiments were subjected to non-linear curve fitting to the equation for a rectangular hyperbola. IC_50_ values for inhibition of [^3^H]imipramine binding by *p*-CA and ibogaine were converted to *K_i_* values using the Cheng-Prusoff approximation (29). Statistically significant differences were verified by analysis of variance or Kruskal-Wallis test followed by the Tukey's or Dunn's multiple comparison post hoc test.

## Author Contributions

M. F. and S. S. designed the experiments and wrote the paper. C. K. and S. S. performed the experiments shown in Figs. 1–3. F. A. E. performed the experiments shown in Figs. 4 and 5. A. E.-K. performed the experiments shown in Fig. 2*e*. W. S. provided Fig. 6 as well as advice and guidance with electrophysiological experiments. All authors reviewed the results and approved the final version of the manuscript.

## Footnotes

3 The abbreviations used are:

5-HT: serotonin (5-hydroxytryptamine)
BetP: betaine transporter (*Corynebacterium glutamicum*)
DAT: dopamine transporter
NET: norepinephrine transporter
*p*-CA: *p*-chloroamphetamine
SERT: serotonin transporter
SLC: solute carrier
PIP_2_: phosphatidylinositol 4,5-bisphosphate
PDL: poly-d-lysine
TBST: TBS with Tween
hSERT: human SERT.
